# Exploring pre-service music teachers' acceptance of generative artificial intelligence: a PLS-SEM-ANN approach

**DOI:** 10.3389/fpsyg.2025.1571279

**Published:** 2025-06-27

**Authors:** Sirui He, Yuhong Ren

**Affiliations:** ^1^Communication University of China, Beijing, China; ^2^Hebei Normal University, Shijiazhuang, Hebei Province, China

**Keywords:** UTAUT2, Generative AI, music education, pre-service teachers, artificial intelligence

## Abstract

**Introduction:**

Based on the extended Unified Theory of Acceptance and Use of Technology Model 2 (UTAUT2), explores the intention to accept Generative Artificial Intelligence (Generative AI) technology in teaching and its influencing factors among pre-service music teachers in higher education.

**Method:**

Quantitative research.

**Results:**

The results indicate that Perceived Risk, Social Influence, and Habit significantly influence Behavioral Intention, while Behavioral Intention and Perceived Risk are key predictors of actual use behavior. Sensitivity analysis further confirms the central role of Behavioral Intention and the inhibitory effect of Perceived Risk.

**Discussion:**

The findings provide theoretical and practical guidance for promoting the application of generative AI in music education.

## 1 Introduction

In recent years, artificial intelligence (AI) has gained increasing prominence in the educational domain, particularly through the adoption of generative AI, which has catalyzed the convergence of traditional pedagogical frameworks with intelligent technologies (Havrilova et al., 2022). While generative AI demonstrates significant potential in music education—transcending conventional resource constraints and enhancing personalized instruction—it simultaneously imposes a discernible cognitive and practical burden on pre-service music educators (Atabek and Burak, 2024). This burden stems from the requisite proficiency in understanding and applying these novel technologies, thereby challenging their preparedness to integrate AI-driven tools into future teaching practices (Atabek and Burak, 2024). In the field of arts education, the application of technology has facilitated the deep integration of traditional education with digital education, opening new pathways for the innovation of teaching methods and tools (Havrilova et al., 2022). Higher education institutions, as key venues for training future music teachers, must constantly monitor technological developments and update their training programs to ensure that pre-service music teachers can master the latest tools and technologies. In teacher training colleges, effective integration of technology and education, as well as curriculum revision, are especially necessary to ensure that pre-service teachers can fully utilize educational technologies once they enter the workforce (Atabek and Burak, 2024). Furthermore, universities should encourage students to engage in self-directed learning and cultivate lifelong learning and skill enhancement awareness and habits, thereby laying a solid foundation for their future careers (Sirek and Sefton, 2024). Through technology, music teachers can more vividly demonstrate music theory, instrumental performance skills, and different musical styles, making abstract concepts more concrete, thus significantly enhancing teaching quality. This concept has been widely recognized among pre-service music teachers, with research indicating that 96.4% of pre-service music teachers believe that technology plays a positive role in music education (Atabek and Burak, 2024). The introduction of technology not only improves classroom efficiency but also provides more opportunities for teacher-student interaction and personalized teaching, helping to optimize teaching outcomes (Pattananon et al., 2024). Empirical studies have further shown that students taught with technology-integrated teaching perform significantly better than those in traditional teaching settings (Lyu and Sokolova, 2023). Therefore, higher education institutions should incorporate technology training into their teaching system when cultivating pre-service music teachers, optimizing teaching outcomes, and enhancing teachers' professional competence. This not only helps music teachers make their classrooms more dynamic, interesting, and modern but also significantly improves teaching effectiveness (Havrilova et al., 2022).

In China, the music curriculum in primary and secondary schools is guided by the Ministry of Education's “National Music Curriculum Standards for Full-time Compulsory Education,” which emphasizes the importance of practical activities, advocates for stimulating students' initiative, and clearly incorporates the “student-centered education philosophy” (Student-Centered Education, SCE). However, due to differences in teachers' understanding of the concept of “educational practice” and the actual teaching environment, there may be discrepancies between teaching implementation and the curriculum standards (Zhang et al., 2023). This is one of the common challenges faced by Chinese music teachers in implementing the SCE philosophy. Due to the large class sizes in public schools, each class typically consists of 38 to 45 students, or even more. Research shows that music teachers often face problems such as uneven time distribution and insufficient classroom time when attempting to implement SCE (Zhang and Leung, 2023). To ensure classroom efficiency and teaching progress, despite the advocacy of SCE in the curriculum standards, teachers often still rely on teacher-centered lectures or even didactic teaching. Therefore, Chinese music classrooms generally lack personalized experiences and exploratory practice. In addition to the macro limitations of large class sizes, teachers also face significant limitations in teaching innovation, especially in technology application. Many in-service music teachers lack relevant training and support for integrating technology into teaching and are still unfamiliar with its practical application in the classroom. Atabek and Burak's (2024) empirical study indicates that although almost all respondents hold a positive attitude toward the use of technology in music classrooms, most teachers believe that its actual use in teaching is still insufficient (Atabek and Burak, 2024). This suggests a clear lack of technological support and pedagogical training in schools (Zhang and Wang, 2024). Therefore, pre-service music teachers need to widely adopt new technologies for student-centered teaching innovation.

With the rapid development of AI, big data, and other cutting-edge technologies, personalized teaching is gradually becoming a reality. In this context, the transformation of teachers' roles and the innovation of teaching methods are particularly critical (Yan and Xia, 2024). For music teachers in mainland China, incorporating generative AI into the classroom can help alleviate teaching pressure, allowing teachers to focus more on student interaction and personalized teaching, thus effectively addressing the current challenges in music education (Wei et al., 2022). Bower et al. (2024) pointed out that teachers generally recognize the positive role of generative AI in supporting teaching practices and optimizing workflows, while also noting its ability to enhance course personalization and address student differences in a timely manner (Bower et al., 2024). By collecting and analyzing students' learning behavior data, generative AI systems can automatically create personalized learning paths for each student, helping teachers identify and resolve teaching challenges arising from individual differences. For students, generative AI can provide immediate feedback in self-directed learning, optimizing traditional learning modes and improving study efficiency (Li and Wang, 2024). This precise guidance model not only enhances learning efficiency but also provides every student with an opportunity to progress at their own pace. For pre-service music teachers, the application of generative AI helps stimulate teaching inspiration and innovation, supporting them in building comprehensive teaching systems during their pre-service phase and focusing more on teaching practice and research, thereby improving their teaching quality (Baratè and Ludovico, 2020; Wei et al., 2022). Therefore, investigating pre-service music teachers' acceptance of generative AI is crucial, as generative AI can better facilitate student-centered teaching innovation.

This study employs the UTAUT2 model proposed by Venkatesh et al. (2012) to explore the acceptance of generative AI technology by pre-service music teachers in higher education. Given the profound impact of advanced technologies on education and the current challenges in Chinese music education, this study focuses on pre-service music teachers in Chinese universities, aiming to understand their intention to use generative AI and the factors influencing this intention. Based on the specific circumstances and context of the research field (Venkatesh et al., 2012), the main goal of this study is to analyze the multiple factors influencing the adoption of generative AI by pre-service music teachers in future teaching, from the perspective of the social group. In the context of generative AI applications in music education, this study expands the UTAUT2 model by integrating factors such as Performance Expectancy, Effort Expectancy, Social Influence, Facilitating Conditions, Hedonic Motivation, Price Value, Habit, Perceived Compatibility, and Perceived Risk. Using PLS-SEM and ANN analysis methods, this study identifies the key factors influencing the future use of generative AI in teaching from the perspective of pre-service music teachers in Chinese universities.

Based on this, the research question of this study is: “How do Performance Expectancy, Effort Expectancy, Social Influence, Facilitating Conditions, Hedonic Motivation, Price Value, Habit, Perceived Compatibility, and Perceived Risk influence the behavioral intention (BI) of pre-service music teachers to use generative AI?”

By elucidating the significant effects of multiple factors on pre-service music teachers' acceptance of generative AI, this study provides empirical evidence for understanding the mechanisms of technology adoption. The findings not only offer guidance for teacher training institutions in designing targeted technology training programs but also provide direction for educational organizations in formulating AI integration strategies, such as optimizing resource support and compatibility design. Additionally, the results inspire technology developers to focus on the specific needs of teaching scenarios, creating intelligent tools that better align with the characteristics of music education. This facilitates the transition of generative AI from being merely technologically feasible to being educationally beneficial.

## 2 Literature review

### 2.1 Research on generative AI in pre-service teacher education

With the development of generative AI, it has shown wide application potential in pre-service teacher education. Noh and Han (2023) noted that pre-service teachers show strong interest in generative AI courses and emphasize that such courses should be practice-oriented to help teachers develop critical analysis skills (Noh and Han, 2023). Kehoe (2023) further explored the value of AI tools in course design, suggesting that these tools can generate high-quality course plans, but still require teachers to adjust them based on their own experiences (Kehoe, 2023). In the field of music education, Mei and Yang (2021) found that pre-service music teachers have a positive attitude toward augmented reality-assisted instrument learning, but still have doubts about its teaching effectiveness (Mei and Yang, 2021). Therefore, despite the significant influence of AI on pre-service teachers, further exploration of their acceptance of technology in music pre-service education is necessary to better prepare them for future high-efficiency teaching. Meanwhile, existing studies on the acceptance of generative AI among pre-service music teachers predominantly adopt macro-level perspectives, focusing on generative AI's role in music pedagogy and its implications for educational policies (Hellman, 2024; Merchán Sánchez-Jara et al., 2024), or conduct experimental investigations into generative AI-assisted teaching tools for specific instruments to examine acceptance levels among teachers and students (Liu and Liao, 2025). Notably, current research lacks systematic exploration of pre-service music teachers as individual agents, particularly regarding their intrinsic acceptance and behavioral determinants toward the technology. This study addresses this critical gap by centering on the subjective perceptions and decision-making processes of pre-service music teachers, thereby enriching the literature on technology adoption in specialized educational contexts.

### 2.2 Research hypotheses

**Figure 1:**
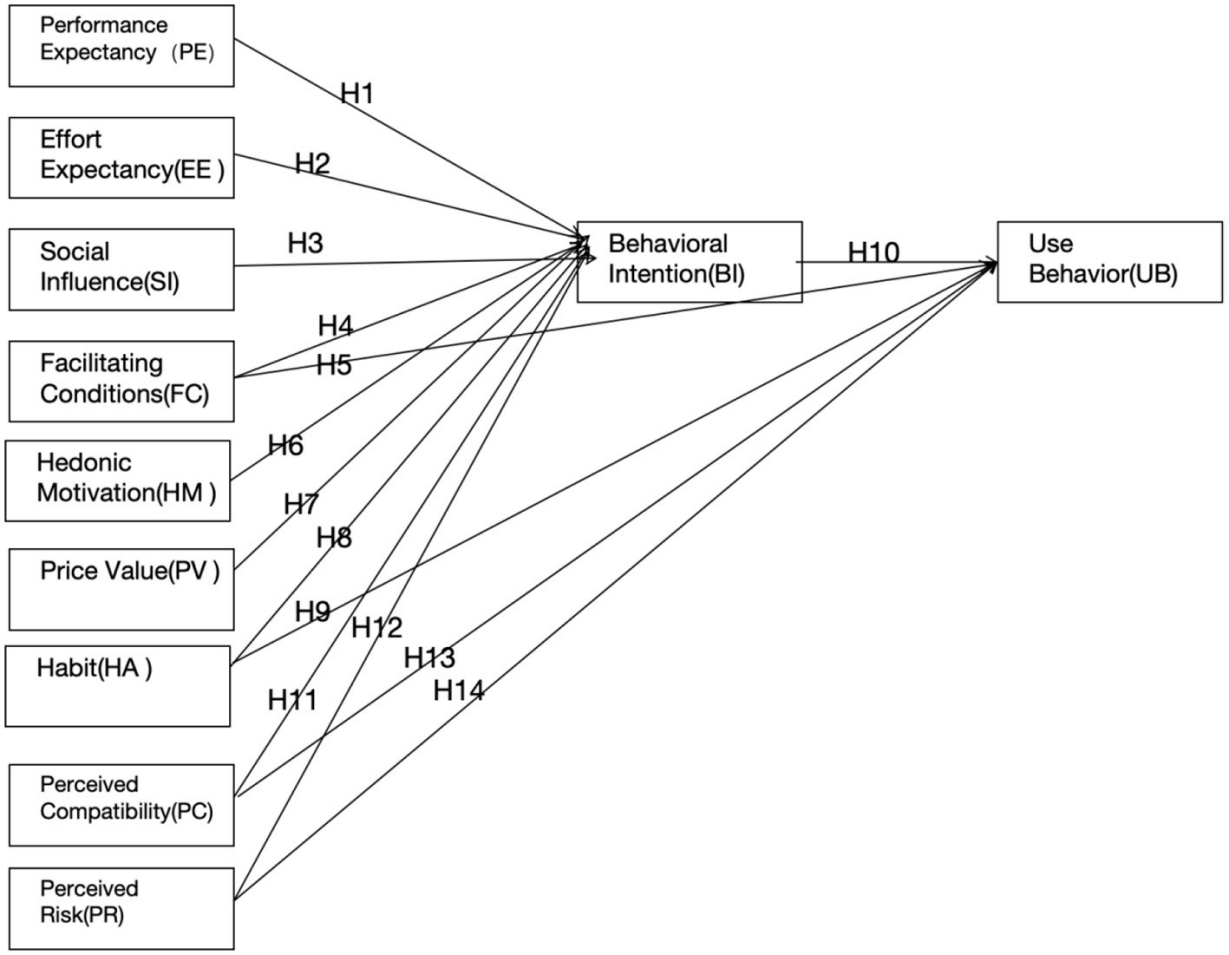
Conceptual framework and research hypotheses.

Based on the UTAUT2 model, this study develops hypotheses and presents the conceptual framework and research hypotheses as shown in Figure 1.

#### 2.2.1 Expanded UTAUT2 model

According to Venkatesh et al. (2012), Performance Expectancy (PE) refers to an individual's expectation of how technology will enhance work performance and improve work conditions. It is one of the key factors predicting Behavioral Intention (BI). In this study, PE specifically refers to pre-service music teachers' attitudes toward generative AI tools and their expected improvements in teaching practice (Venkatesh et al., 2012; Wijaya et al., 2024). Effort Expectancy (EE) represents users' perceived ease of use of new technology, and it is also an important variable influencing BI (Venkatesh et al., 2003, 2012). Social Influence (SI) refers to the effect that significant others have on the user's attitude toward using the technology, which plays a critical role in BI prediction (Venkatesh et al., 2003, 2012). Facilitating Conditions (FC) refer to users' perceptions of the availability of technical support, resources, and training, which directly impact both BI and actual usage behavior (UB) (Ruiz-Rojas et al., 2023; Venkatesh et al., 2003). Additionally, Hedonic Motivation (HM), the pleasure derived from using the technology, is believed to significantly influence BI (Choi et al., 2023; Venkatesh et al., 2012; Yang and Appleget, 2024). Price Value (PV) measures users' perceptions of the cost-effectiveness of technology, and when users perceive that the cost is proportional to the benefits, BI is strengthened (Venkatesh et al., 2012; Alhwaiti, 2023). Lastly, Habit (HT), the tendency of users to use technology automatically, has a significant impact on both BI and UB (Venkatesh et al., 2012; Walker et al., 2020; Xu et al., 2024). Based on these theories, this study proposes the following hypotheses:

H1: Performance Expectancy (PE) has a positive influence on pre-service music teachers' Behavioral Intention (BI) to use generative AI tools.
H2: Effort Expectancy (EE) has a positive influence on pre-service music teachers' Behavioral Intention (BI) to use generative AI tools.
H3: Social Influence (SI) has a positive influence on pre-service music teachers' Behavioral Intention (BI) to use generative AI tools.
H4: Facilitating Conditions (FC) have a positive influence on pre-service music teachers' Behavioral Intention (BI) to use generative AI tools.
H5: Facilitating Conditions (FC) have a positive influence on pre-service music teachers' Actual Usage Behavior (UB) of generative AI tools.
H6: Hedonic Motivation (HM) has a positive influence on pre-service music teachers' Behavioral Intention (BI) to use generative AI tools.
H7: Price Value (PV) has a positive influence on pre-service music teachers' Behavioral Intention (BI) to use generative AI tools.
H8: Habit (HT) has a positive influence on pre-service music teachers' Behavioral Intention (BI) to use generative AI tools.
H9: Habit (HT) has a positive influence on pre-service music teachers' Actual Usage Behavior (UB) of generative AI tools.
H10: Behavioral Intention (BI) has a positive influence on pre-service music teachers' Actual Usage Behavior (UB) of generative AI tools.

The expanded variables selected in this study are Perceived Compatibility and Perceived Risk. Perceived Compatibility refers to users' perception of how consistent the new technology is with their existing tools and practices. In this study, Perceived Compatibility describes how pre-service music teachers perceive the compatibility of generative AI with their current teaching methods and tools. If teachers perceive the new technology as highly compatible with existing teaching systems and methods, they are more likely to adopt it. Perceived Risk refers to teachers' concerns about the potential risks of using generative AI, such as information security, student privacy, and data safety. These perceived risks may affect whether they are willing to introduce AI technology into their classrooms (Zhang et al., 2024). Based on this, the study proposes the following hypotheses:

H11: Perceived Compatibility (PC) has a positive influence on pre-service music teachers' Behavioral Intention (BI) to use generative AI tools.
H12: Perceived Compatibility (PC) has a positive influence on pre-service music teachers' Actual Usage Behavior (UB) of generative AI tools.
H13: Perceived Risk (PR) has a negative influence on pre-service music teachers' Behavioral Intention (BI) to use generative AI tools.
H14: Perceived Risk (PR) has a negative influence on pre-service music teachers' Actual Usage Behavior (UB) of generative AI tools.

## 3 Method

### 3.1 Sample selection and data collection

The study strictly adhered to the ethical standards outlined in the 1964 Helsinki Declaration and its subsequent amendments or comparable ethical standards. Researchers provided informed consent forms to all participants, and all participants voluntarily took part in the study. The study was conducted anonymously, and participants had the right to withdraw from the study at any time. The sample comprised 301 pre-service music teachers from various music conservatories across the country. Purposive sampling and snowball sampling methods were employed to ensure regional representativeness. Given that the target group for this study consists of pre-service music teachers, samples were screened based on certain criteria: all participants were students enrolled in music education programs, had some teaching experience, and planned to pursue a career in music education in the future, ensuring that their attitudes and behaviors in the survey were both representative and credible. The “currently enrolled” criterion is based on the “pre-service” nature of the group, and by narrowing the sample to students, the study can explore the acceptance of generative AI technology within the specific context of music education. The condition of “having some teaching experience” ensures that participants possess relevant practical knowledge when completing the questionnaire, which leads to more accurate data. The “music education career planning” condition was set to better understand the influence of multiple factors on the participants' behavioral intentions and usage behaviors, and to provide a more comprehensive view of the current and future potential of generative AI in music education.

The participants ranged from undergraduate freshmen to third-year graduate students, aiming to cover a broad spectrum of pre-service music teachers at different stages of learning and practice. The researchers conducted a pilot study involving 30 participants to test the reliability of the survey instrument. The results indicated a Cronbach's alpha value of 0.91, demonstrating that the questionnaire is reliable. The data collection started on October 21, 2024, and lasted for 1 week. An online survey was distributed via the Wenjuanxing platform and QR codes were shared in WeChat groups of music education programs across various institutions. The questionnaire used a five-point Likert scale (1 = strongly disagree, 5 = strongly agree), ensuring anonymity and confidentiality to protect participants while encouraging active participation in the study.

The researchers conducted a power analysis using G^*^Power software, based on the expected effect size, significance level (usually 0.05), and the required statistical power (typically 0.80), and determined that a sample size of 301 participants is sufficient. A total of 301 valid questionnaires were collected. This study contains no missing data, as the survey questionnaire was designed to require responses to all questions before submission. The researchers retained outliers in the dataset. Among the 301 respondents, 103 were male (34%) and 198 were female (66%), with the majority being aged between 18 and 21. The sample included 31 freshmen (10.2%), 45 sophomores (15%), 105 juniors (34.9%), 96 seniors (31.9%), and 24 graduate students (8%). The time spent completing the questionnaire ranged from 5 min to 20 min.

### 3.2 Measurement instruments

The questionnaire used in this study was divided into two sections. The first section collected participants' basic information, including age, grade level, teaching experience, and gender, to filter out non-eligible samples. The second section combined the UTAUT model with technology acceptance variables, including Performance Expectancy, Effort Expectancy, Social Influence, Facilitating Conditions, Hedonic Motivation, Price Value, Habit, Perceived Compatibility, Perceived Risk, Behavioral Intention (BI), and Usage Behavior (UB). A reverse-item question was included to identify invalid responses, ensuring the data used in the analysis was reliable. A detailed list of the items used in the study is provided in Appendix 1.

### 3.3 Data analysis

This study used both PLS-SEM and Artificial Neural Networks (ANN) methods to test the research hypotheses and construct a predictive model for pre-service music teachers' behavior toward generative AI tools. PLS-SEM was chosen because it can handle small sample sizes with non-normal distributions and is suitable for constructing and analyzing predictive models (Hair Jr. et al., 2021). ANN was selected because it can overcome the limitations of non-compensatory models and capture both linear and nonlinear relationships, thereby improving predictive accuracy (Ali et al., 2025; Sharma et al., 2021; Soliman et al., 2024; Hair et al., 2012). Moreover, due to its “black-box” nature, ANN is more suitable for prediction rather than hypothesis testing, providing more accurate results for the study. The configuration of the Artificial Neural Network (ANN) is specified as a multi-layer perceptron (MLP) consisting of three layers, with the input layer containing 9 neurons (corresponding to 9 input features) and the output layer containing 1 neuron (corresponding to the prediction result). In the hidden layer, 10 neurons can be set. The hidden layer can use the ReLU (Rectified Linear Unit) activation function to enable the model to learn non-linear relationships.

## 4 Data analysis

### 4.1 Measurement (outer) model evaluation

Convergent validity refers to the degree of correlation between two constructs that are theoretically expected to be related (Strauss and Smith, 2009). This study presents key reliability and validity indices, including Cronbach's alpha, Composite Reliability, and Average Variance Extracted (AVE), in Table 1. Reliability is assessed based on the measurement values of Cronbach's alpha and Composite Reliability. The Harman's single-factor test was employed to assess common method variance (CMV). The results demonstrated that no single factor accounted for a majority of the variance, indicating that CMV was not a primary concern in this study. According to the data in Table 1, the Cronbach's alpha values range from 0.776 to 0.871, all significantly exceeding the recommended threshold of 0.7, indicating good internal consistency of the scales. Additionally, the minimum value of Composite Reliability is 0.777, which also meets the recommended threshold of 0.7. This demonstrates that the convergent validity of the constructs in this study is supported. Furthermore, the AVE values in Table 1 range from 0.691 to 0.795, all exceeding the minimum threshold of 0.5, further validating the convergent validity of the constructs. In conclusion, all measurement indices meet the standards set by Hair et al. (2017), indicating that the reliability and validity of this study are at an ideal level.

**Table 1:** Cronbach's alpha, composite reliability, and average variance extracted (AVE).

**Variable**	**Cronbach's alpha**	**Composite reliability (rho_a)**	**Composite reliability (rho_c)**	**Average variance extracted (AVE)**
BI	0.871	0.871	0.921	0.795
EE	0.781	0.787	0.872	0.694
FC	0.842	0.846	0.905	0.761
HA	0.806	0.807	0.886	0.721
HM	0.801	0.806	0.883	0.715
PC	0.8	0.809	0.882	0.714
PE	0.816	0.818	0.891	0.731
PR	0.816	0.818	0.891	0.731
PV	0.776	0.777	0.87	0.691
SI	0.84	0.857	0.904	0.758
UB	0.819	0.821	0.893	0.735

Discriminant validity measures the degree of independence between variables. Table 2 presents the Heterotrait-Monotrait Ratio (HTMT) values between constructs. HTMT is a stricter measure of discriminant validity, used to assess the degree of similarity between different constructs. According to the literature, HTMT values should be below 0.85, or ideally 0.90, to demonstrate adequate discriminant validity (Henseler et al., 2015).

**Table 2:** Discriminant validity—Heterotrait-monotrait ratio (HTMT).

**Variable**	**BI**	**EE**	**FC**	**HA**	**HM**	**PC**	**PE**	**PR**	**PV**	**SI**	**UB**
BI											
EE	0.596										
FC	0.629	0.463									
HA	0.636	0.386	0.444								
HM	0.636	0.411	0.393	0.445							
PC	0.573	0.435	0.368	0.344	0.344						
PE	0.63	0.411	0.446	0.377	0.45	0.457					
PR	0.686	0.466	0.486	0.366	0.414	0.388	0.457				
PV	0.663	0.397	0.391	0.384	0.447	0.379	0.52	0.533			
SI	0.551	0.237	0.313	0.441	0.366	0.251	0.379	0.407	0.379		
UB	0.816	0.523	0.598	0.625	0.544	0.616	0.588	0.654	0.571	0.425	

### 4.2 Structural (inner) model evaluation

#### 4.2.1 Collinearity statistics measurement - variance infation factors (VIF)

To test whether collinearity is an issue in the model, this study conducted a collinearity statistics measurement. All VIF values are below 5, with the minimum being 1.575 for EE3 and the maximum being 2.976 for BI1 (Hair Jr. et al., 2021), indicating no significant multicollinearity issues in the model. This indicates that there is no multicollinearity issue among the variables in the model, confirming the validity of the model design.

#### 4.2.2 Path coefficient (β value)

**Figure 2:**
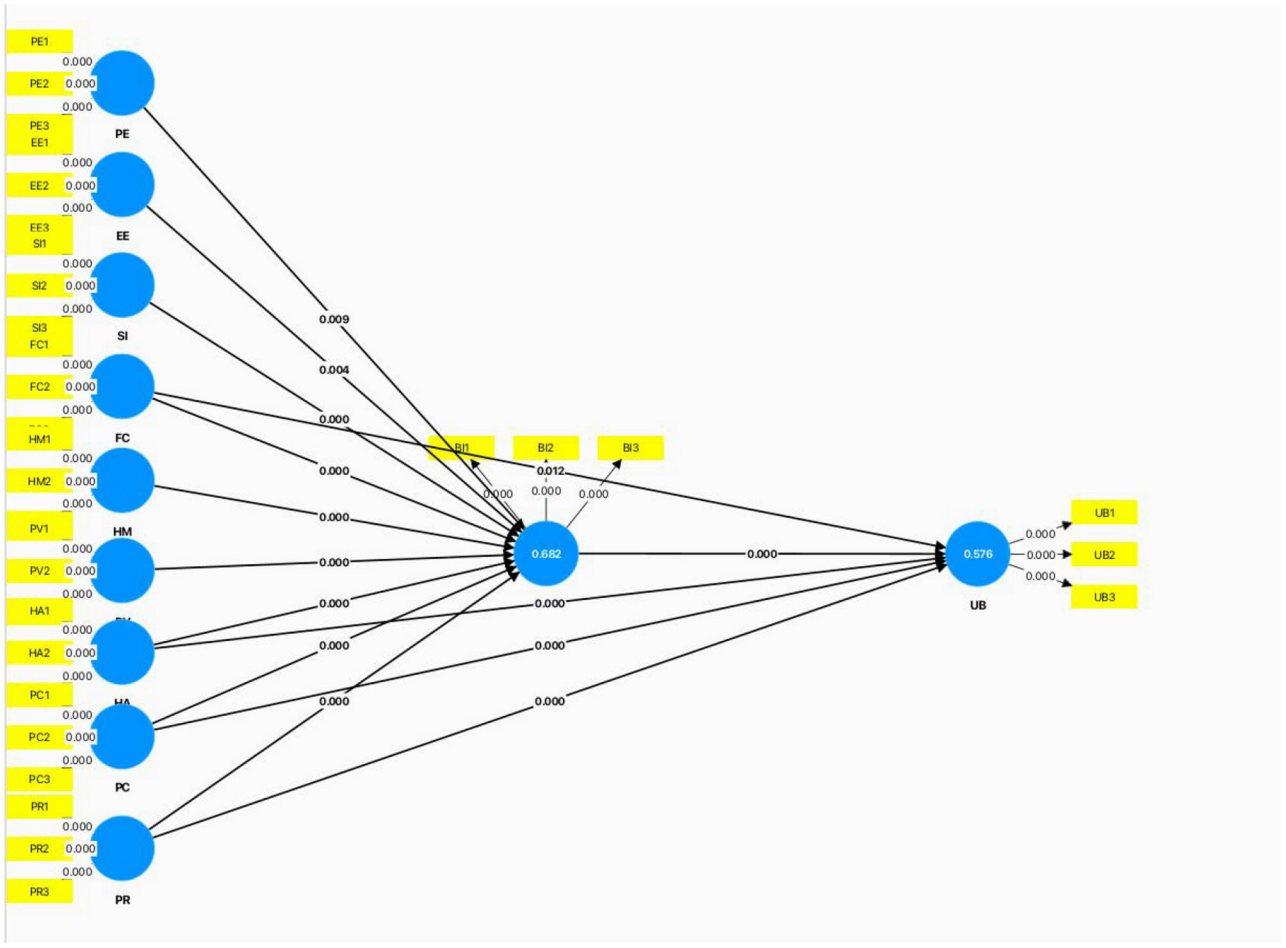
Confirmatory model results.

According to the results in Table 3 and Figure 2, all hypothesized paths were significantly supported. First, Behavioral Intention (BI) has a significant positive impact on Actual Usage Behavior (UB) (path coefficient = 0.327, t-statistic = 5.598, *p* < 0.001). In addition, Performance Expectancy (EE), Facilitating Conditions (FC), and Habit (HA) all have significant positive influences on Behavioral Intention (BI), with FC and HA having stronger effects (path coefficients of 0.145 and 0.168, respectively, *p* < 0.05). Regarding Actual Usage Behavior (UB), Perceived Compatibility (PC) has the most significant impact (path coefficient = 0.203, *t*-statistic = 4.909, *p* < 0.001), followed by Habit (HA) and Perceived Risk (PR), both of which have significant effects on UB (path coefficients of 0.179 and 0.178, respectively, *p* < 0.05).

**Table 3:** Results of hypotheses.

**Variable**	**Original sample (O)**	**Sample mean (M)**	**Standard deviation (STDEV)**	**T statistics (|O/STDEV|)**	***P*-values**
BI → UB	0.327	0.326	0.058	5.598	0.000
EE → BI	0.11	0.11	0.038	2.884	0.004
FC → BI	0.145	0.145	0.04	3.629	0.000
FC → UB	0.124	0.124	0.049	2.526	0.012
HA → BI	0.168	0.169	0.035	4.882	0.000
HA → UB	0.179	0.18	0.049	3.693	0.000
HM → BI	0.155	0.155	0.041	3.775	0.000
PC → BI	0.135	0.135	0.037	3.613	0.000
PC → UB	0.203	0.204	0.041	4.909	0.000
PE → BI	0.11	0.112	0.042	2.631	0.009
PR → BI	0.182	0.182	0.043	4.198	0.000
PR → UB	0.178	0.178	0.048	3.739	0.000
PV → BI	0.154	0.153	0.042	3.647	0.000
SI → BI	0.134	0.134	0.038	3.51	0.000

#### 4.2.3 Variance (r^2^)

The R^2^ value for Behavioral Intention (BI) is 0.682, and the *R*^2^ value for Actual Usage Behavior (UB) is 0.576. This indicates that the independent variables in the model can explain 68.2% of the variance in Behavioral Intention and 57.6% of the variance in Actual Usage Behavior. The higher *R*^2^ value for Behavioral Intention suggests that the model has a strong predictive effect on BI. Although the *R*^2^ value for UB is slightly lower, it still indicates that the model has moderate explanatory power for UB.

#### 4.2.4 Predictive relevance (Q^2^)

The Q^2^ value for Behavioral Intention (BI) is 0.526, and the Q^2^ value for Actual Usage Behavior (UB) is 0.405. The Q^2^ value is a key criterion for assessing a model's predictive relevance, and a Q^2^ greater than 0 indicates that the model has predictive relevance. Specifically, the Q^2^ value for BI (0.526) indicates that the model has strong predictive ability for BI, while the Q^2^ value for UB (0.405) shows moderate predictive relevance. According to Hair et al. (2017), a Q^2^ value exceeding 0.35 is considered to have moderate predictive ability, thus the model demonstrates a satisfactory predictive performance for both Behavioral Intention and Actual Usage Behavior.

#### 4.2.5 Measurement of effect size (f2) and (q2)

The *f*^2^ values of the constructs in the model reflect the relative contribution of the independent variables to explaining the dependent variables. *f*^2^ values of 0.02, 0.15, and 0.35 are considered to represent small, medium, and large effects, respectively. Table 4 shows the *f*^2^ values of the constructs for Behavioral Intention (BI) and Actual Usage Behavior (UB). The *f*^2^ values for BI ranged between 0.026 and 0.069, while those for UB ranged between 0.025 and 0.107. This measurement indicates the explanatory power of each predictor. Table 5 presents the q^2^ values, which reflect the incremental predictive contribution of each variable to the endogenous constructs. Following the recommendations of Hair et al. (2017), *q*^2^ values greater than 0 indicate predictive relevance, while values below 0 indicate no predictive relevance. Generally, *q*^2^ values above 0.02 (small effect), 0.15 (medium effect), and 0.35 (large effect) are considered significant. The results of *f*^2^ and *q*^2^ for this study are provided in Tables 6, 7.

**Table 4:** Full model *f*^2^.

**Variable**	**BI**	**EE**	**FC**	**HA**	**HM**	**PC**	**PE**	**PR**	**PV**	**SI**	**UB**
BI	/	0.028	0.047	0.065	0.055	0.044	0.026	0.069	0.052	0.043	/
UB	0.107	/	0.025	0.053	/	0.074	/	0.049	/	/	/

**Table 5:** q^2^.

**Variable**	**EE**	**FC**	**HA**	**HM**	**PC**	**PE**	**PR**	**PV**	**SI**
BI	0.0087	0.0173	0.0229	0.0187	0.0130	0.0087	0.0229	0.0173	0.0158
UB	0.0000	0.0058	0.0133	0.0000	0.0180	0.0000	0.0086	0.0000	0.0000

**Table 6:** RMSE values for error.

**Variable**	**Model A**		**Model B**	
	**Input: PE, EE, SI, FC, HM, PV, HA, PC, PR**		**Input: FC, BI, HA, PC, PR**	
	**Ouput: BI**		**Ouput: UB**	
**Neural network**	**Training**	**Testing**	**Training**	**Testing**
ANN1	0.168	0.170	0.217	0.200
ANN2	0.194	0.014	0.249	0.159
ANN3	0.166	0.141	0.242	0.221
ANN4	0.180	0.208	0.226	0.155
ANN5	0.151	0.179	0.244	0.198
ANN6	0.225	0.124	0.189	0.345
ANN7	0.154	0.199	0.213	0.219
ANN8	0.170	0.134	0.233	0.253
ANN9	0.201	0.181	0.208	0.241
ANN10	0.175	0.084	0.205	0.267
Mean	0.178	0.143	0.223	0.226
SD	0.150	0.243	0.139	0.236
	0.023	0.059	0.019	0.056

**Table 7:** Sensitivity analysis.

**Variable**	**Model A(output: BI)**									**Model B(output: UB)**				
**Neural network**	**PE**	**EE**	**SI**	**FC**	**HM**	**PV**	**HA**	**PC**	**PR**	**HA**	**PC**	**PR**	**BI**	**FC**
ANN1	0.082	0.031	0.172	0.136	0.100	0.054	0.118	0.200	0.106	0.143	0.180	0.184	0.330	0.162
ANN2	0.058	0.032	0.191	0.130	0.141	0.068	0.053	0.142	0.185	0.340	0.174	0.313	0.137	0.036
ANN3	0.138	0.085	0.104	0.115	0.109	0.109	0.133	0.093	0.115	0.131	0.248	0.261	0.191	0.170
ANN4	0.145	0.074	0.140	0.077	0.165	0.044	0.130	0.089	0.136	0.167	0.208	0.203	0.264	0.157
ANN5	0.080	0.103	0.115	0.103	0.121	0.125	0.142	0.079	0.133	0.165	0.236	0.097	0.300	0.202
ANN6	0.055	0.288	0.149	0.019	0.064	0.059	0.112	0.105	0.148	0.227	0.225	0.198	0.279	0.070
ANN7	0.061	0.079	0.115	0.126	0.128	0.150	0.120	0.090	0.132	0.129	0.177	0.195	0.397	0.102
ANN8	0.064	0.084	0.088	0.189	0.121	0.122	0.090	0.092	0.150	0.095	0.228	0.283	0.190	0.204
ANN9	0.066	0.103	0.161	0.105	0.047	0.182	0.205	0.069	0.063	0.162	0.178	0.162	0.360	0.137
ANN10	0.169	0.075	0.094	0.160	0.128	0.102	0.139	0.075	0.058	0.172	0.194	0.179	0.347	0.108
Average relative imporance	0.092	0.095	0.133	0.116	0.112	0.102	0.124	0.103	0.123	0.173	0.205	0.208	0.280	0.135
Normanlized relative importance (%)	69.023	71.729	100.000	87.218	84.511	76.316	93.383	77.744	92.180	61.821	73.143	74.107	100.000	48.143

### 4.3 Artificial neural network analysis

**Figure 3:**
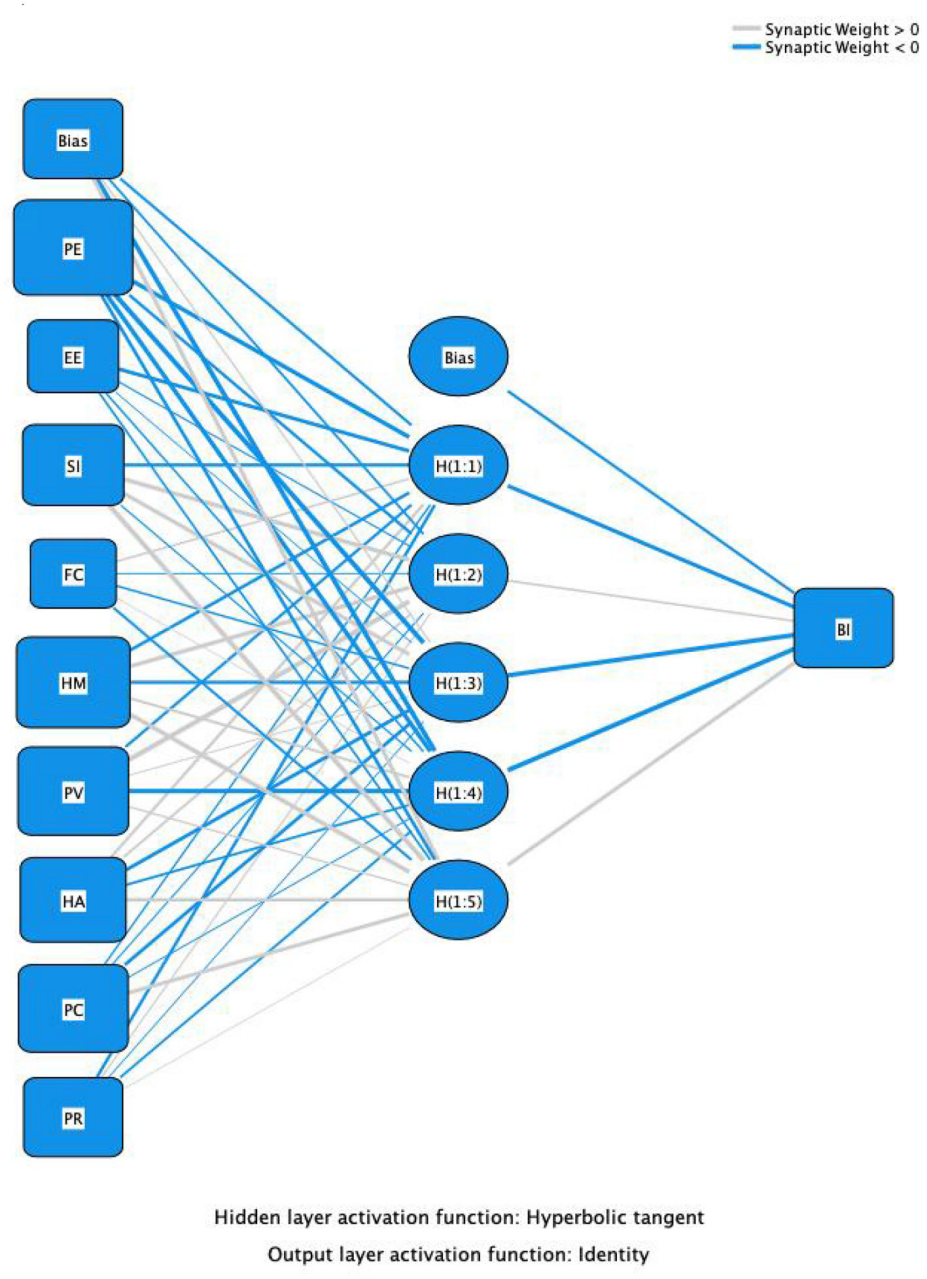
Artificial neural network model construction – BI.

**Figure 4:**
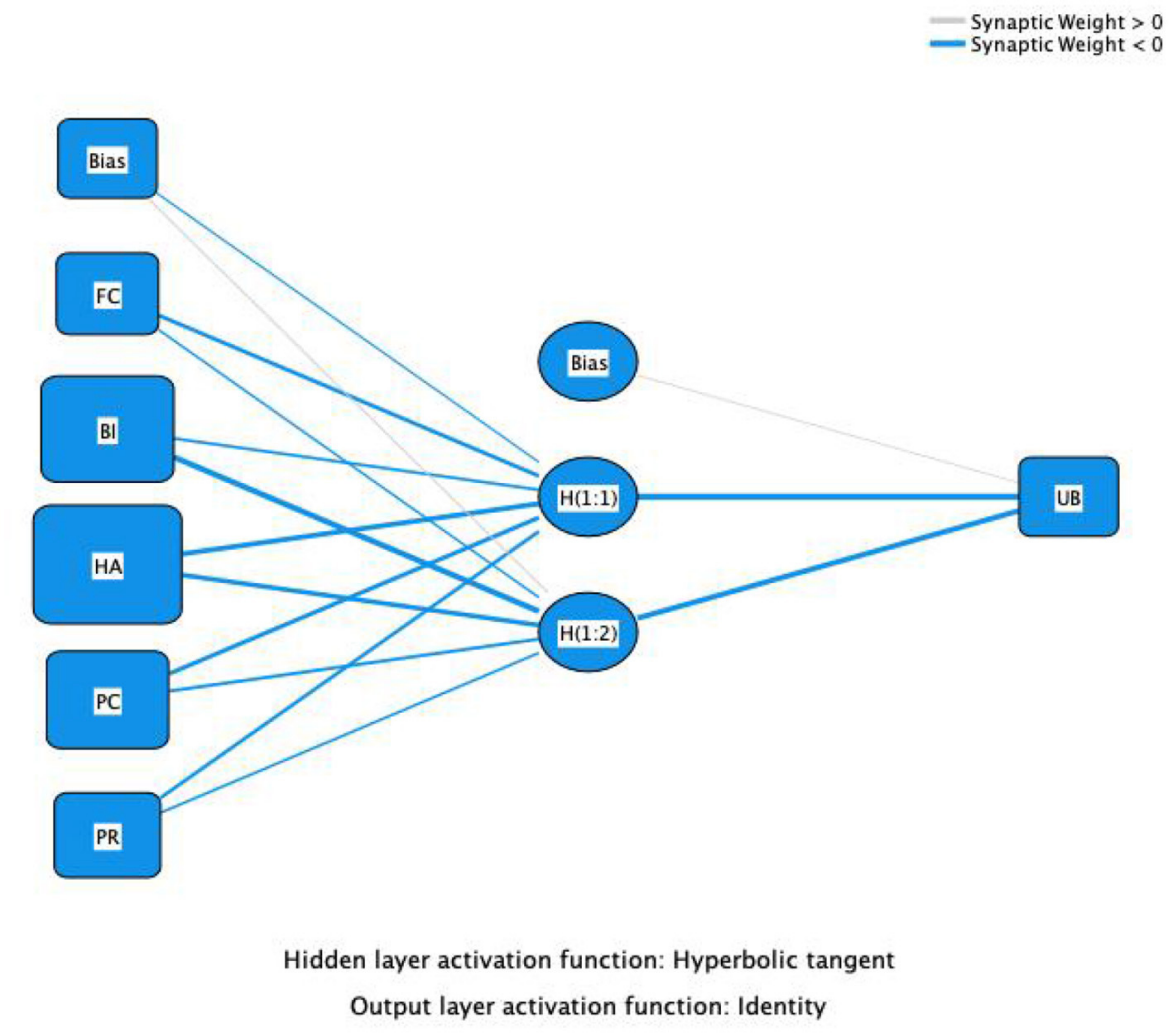
Artificial neural network model construction – UB.

In this study, key influential factors identified through PLS-SEM path analysis were used as input neurons for the Artificial Neural Network (ANN), as shown in Figures 3, 4. The samples were divided into a 90:10 ratio for training and testing purposes. To minimize errors, a 10-fold cross-validation using Root Mean Square Error (RMSE) was applied.

As shown in Table 6, the RMSE mean for Model A during the testing phase was 0.143, with a standard deviation (SD) of 0.059. The testing error was slightly lower than the training error, indicating that the model performed well in terms of generalization and did not exhibit overfitting. For Model B, the RMSE mean during the testing phase was 0.226, with an SD of 0.056. The testing error was slightly higher than the training error, suggesting that the model's performance on the test data was similar to its performance on the training data, indicating good generalization ability.

As shown in Table 7, in Model B, the most prominent predictor of Actual Usage Behavior (UB) was Behavioral Intention (BI), with a normalized importance of 100%, indicating that it was the most critical factor influencing UB. Following BI, Perceived Risk (PR, 74.11%) and Facilitating Conditions (FC, 48.14%) were the next most important factors. While Perceived Compatibility (PC) and Habit (HA) had relatively lower importance, they still exerted some influence. In Model A, Social Influence (SI) had the largest predictive contribution to Behavioral Intention (BI) (average relative importance: 0.133), normalized to 100%, indicating its central position in the model. The next most important factors were Habit (HA, 93.38%) and Facilitating Conditions (FC, 87.22%).

## 5 Discussion

### 5.1 Findings interpretation

First, the research results indicate that performance expectancy has a significant positive effect on the pre-service music teachers' usage behavior (H1). Pre-service music teachers in China believe that if AI tools can enhance teaching efficiency and reduce lesson preparation difficulties, they will be more willing to use generative AI tools in their future teaching. This may be related to the current educational challenges in China, as appropriate teaching aids can alleviate teachers' workload. Additionally, such aids play a positive role across various subjects, assisting teachers in class planning while also creating interactive classroom environments that support adaptive learning processes (Zhu and Yang, 2023). Furthermore, when pre-service music teachers perceive that they can easily master generative AI technology, their intention to use the technology increases accordingly (H2). Shahzad et al. (2024) demonstrated that the ease of use of generative AI positively influences technology acceptance and usage behavior, which aligns with the results of this study (Shahzad et al., 2024).

In addition, the data from this study support the positive influence of social influence on the acceptance of generative AI technology by pre-service music teachers (H3). This result also confirms Wang et al. (2024)'s view that the influence of those around them affects pre-service teachers' use of AI tools (Zhang and Wang, 2024). In this study, based on feedback from pre-service music teachers at surveyed universities, professional teachers have gradually integrated generative AI into their classrooms. When pre-service music teachers observe the teaching advantages brought by AI through their students' perspectives, their intention to use the technology increases. Similarly, the surveyed universities have implemented measures such as AI training, advocating for technological awareness, and teacher guidance to provide the conditions for pre-service teachers to learn the technology, thus encouraging the willingness of music education students to use generative AI.

Fourth, the hypotheses concerning the influence of facilitating conditions (FC) on usage behavior and intention (H4, H5) were also supported. Venkatesh et al. (2003) confirmed the impact of FC on UB, and the data from this study further support this classic conclusion.

Fifth, the study of hedonic motivation examined the relationship between the pre-service music teachers' experience of using generative AI and their intention to use it. The results show that when pre-service music teachers find enjoyment in using the technology, their willingness and behavior to use it in the future will increase (H6). This suggests that the acceptance of technology depends on users' positive psychological changes, such as reduced work pressure, which motivates them to use the technology (Chiu et al., 2024). These findings directly correspond to H8 and H9, indicating that habitual use of generative AI technology has a positive effect on overall usage intention. Similarly, the data from this study support the view that when the price of the technology is considered worth its value, pre-service music teachers' willingness to use the technology increases (H7). The findings from this study further validate Alhwaiti's (2023) view that when generative AI demonstrates good usability, teachers are more willing to pay for it (Alhwaiti, 2023).

The two new factors introduced in this study—Perceived Compatibility (PC) and Perceived Risk (PR)—were also found to have a positive feedback effect on pre-service music teachers' usage intention and behavior toward generative AI (H11, H12, H13, H14). Regarding perceived compatibility, generative AI needs to have a high degree of compatibility with existing teaching models, facilitating a balance between new and old technologies to avoid increased time costs due to the need to adapt to the new technology. The data indicate that the pre-service music teachers surveyed believe that generative AI technology can be compatible with current teaching practices and has the potential to enhance the teaching model (Luckin and Holmes, 2016).

### 5.2 Practical implications

The hypotheses and conclusions proposed in this study explore the current application of advanced technology in the field of education and the corresponding user attitudes toward it, while also revealing the positive impact of generative AI on music education teaching practices and development. The feedback from pre-service music teachers on their acceptance of generative AI can guide higher education institutions in updating their training programs and designing more effective, targeted courses. This can help alleviate some of the resistance and misconceptions pre-service music teachers may have toward technology, ultimately optimizing curriculum design. This study also provides a research case for interdisciplinary educational research. By combining education, music, and computer science, the study of pre-service music teachers offers a typical advanced conceptual case for interdisciplinary teaching, demonstrating the integration and interaction of knowledge and skills from different disciplines in educational practice. This can serve as a reference for other educational disciplines in exploring technology acceptance.

### 5.3 Theoretical implications

The positive feedback from performance expectancy provides new directions for innovation in music education, highlighting the application and development potential of generative AI in music teaching. Especially in the context of Chinese society, this study is expected to promote the transformation of the traditional music education model, which remains dominant in China, toward a more digitalized and personalized teaching approach. Moreover, through personalized AI-assisted teaching, it can promote the practical and effective implementation and popularization of Student-Centered Education (SCE) in China. Second, for the pre-service music teacher group, the results of this study reflect the current inadequacies in higher education's training programs in addressing the rapidly evolving new technologies. Higher education institutions can optimize their training programs and resource allocation based on the findings of this study to enhance their advanced teaching standards and help pre-service music teachers acquire sufficient technological skills before entering the workforce.

### 5.4 Limitations and future research

Although this study has certain research significance, it still has some limitations. The 301 questionnaire surveys collected from various Chinese higher education institutions did not strictly limit or categorize regions. Given China's vast territory, there may be regional differences in music education levels and the acceptance and emphasis on emerging technologies. Additionally, the study did not differentiate between the types of higher education institutions (e.g., music conservatories, comprehensive universities, normal universities), which could lead to differences in training programs, teaching philosophies, and teaching objectives, potentially affecting the generalizability of the data.

This research direction also has sustainability. In the future, as generative AI technology becomes widely accepted in music education, research on the integration of music education and generative AI technology can shift from technology mastery and acceptance to an in-depth exploration of how technology-integrated teaching provides personalized, contextual feedback in real-time for students. Research could investigate whether such a technology-integrated educational model has a positive effect on the creativity of young learners and whether it helps students express themselves better in their learning. This represents not only personalized learning but also offers certain protection and enhancement of divergent thinking in children and adolescents. When generative AI becomes more prevalent in the music education field, future researchers may attempt to design a set of evaluation criteria to more specifically assess the teaching-assisted effects of generative AI, including long-term monitoring of student performance metrics, interest indicators, and teacher performance indicators. This would transition the study from quantitative research to qualitative research.

The application of technology inevitably generates data. To ensure the long-term effectiveness of generative AI assistance, memory functions should be incorporated. However, with the widespread application of generative AI, large amounts of feedback data will be stored. Future research could focus on how to ensure the secure storage of data and protect students' privacy to prevent data leakage or misuse. Additionally, ethical issues surrounding the use of generative AI in teaching, such as academic integrity, deserve further exploration. Educators should be guided on how to appropriately teach students to approach generative AI with the correct attitude, avoiding ethical issues like academic dishonesty.

## 6 Conclusion

This study, based on the extended UTAUT2 model, explores the acceptance intention and influencing factors of pre-service music teachers in Chinese universities regarding the use of generative AI in teaching. By introducing the new variables of Perceived Compatibility (PC) and Perceived Risk (PR), the study enhances the applicability of the model in specific contexts. The study employed PLS-SEM and ANN methods for hypothesis testing and predictive analysis, comprehensively assessing the impact of various factors on Behavioral Intention (BI) and Actual Usage Behavior (UB). The findings offer practical guidance for optimizing technology acceptance and usage strategies among pre-service music teachers.

## Data Availability

The original contributions presented in the study are included in the article/Supplementary material, further inquiries can be directed to the corresponding author.
