# Supplementary material for: Exploring pre-service music teachers' acceptance of generative artificial intelligence: a PLS-SEM-ANN approach
Source: Front Psychol. 2025 Jun 27;16:1571279. doi: 10.3389/fpsyg.2025.1571279 (PMC12247848; doi:10.3389/fpsyg.2025.1571279)
Supplement: Supplementary file 1 [file Data_Sheet_1.docx]

**Supplementary Information**

**Supplementary Note 1: Questionnaire**

**Pre-service Music Teachers' Survey on the Use of Generative AI Technology**

Performance Expectancy

1.1 I believe that generative AI technology can improve my teaching effectiveness.

1.2 I believe that using generative AI technology allows me to complete teaching tasks more efficiently.

1.3 Generative AI technology helps to enhance the quality of my teaching.

(1 = Strongly Disagree, 2 = Disagree, 3 = Neutral, 4 = Agree, 5 = Strongly Agree)

Effort Expectancy

2.1 I believe that generative AI technology is easy to learn and use.

2.2 I think the effort required to master generative AI technology is reasonable.

2.3 Learning how to use generative AI technology is simple for me.

(1 = Strongly Disagree, 2 = Disagree, 3 = Neutral, 4 = Agree, 5 = Strongly Agree)

Social Influence

3.1 My colleagues/advisors believe that I should use generative AI technology.

3.2 My peers support my use of generative AI technology.

3.3 I feel external pressure (e.g., from colleagues or friends) to use generative AI technology.

(1 = Strongly Disagree, 2 = Disagree, 3 = Neutral, 4 = Agree, 5 = Strongly Agree)

Facilitating Conditions

4.1 I have the necessary resources (e.g., equipment, software) to use generative AI technology.

4.2 My working environment supports the use of generative AI technology.

4.3 I am able to obtain the necessary help to use generative AI technology.

(1 = Strongly Disagree, 2 = Disagree, 3 = Neutral, 4 = Agree, 5 = Strongly Agree)

Hedonic Motivation

5.1 I find using generative AI technology enjoyable.

5.2 Using generative AI technology makes me feel happy.

5.3 I think using generative AI technology is an enjoyable experience.

(1 = Strongly Disagree, 2 = Disagree, 3 = Neutral, 4 = Agree, 5 = Strongly Agree)

Price Value

6.1 I think generative AI technology offers good value for the cost.

6.2 The cost of using generative AI technology is reasonable.

6.3 I think the overall value of generative AI technology is worth it for me.

(1 = Strongly Disagree, 2 = Disagree, 3 = Neutral, 4 = Agree, 5 = Strongly Agree)

Habit

7.1 I am accustomed to using generative AI technology in my daily teaching.

7.2 Using generative AI technology has become a part of my daily work.

7.3 I tend to rely on generative AI technology in my teaching.

(1 = Strongly Disagree, 2 = Disagree, 3 = Neutral, 4 = Agree, 5 = Strongly Agree)

Perceived Compatibility

8.1 I believe that generative AI technology is compatible with my teaching methods.

8.2 Generative AI technology is suitable for use in my teaching content.

8.3 I believe that generative AI technology aligns with my teaching goals.

(1 = Strongly Disagree, 2 = Disagree, 3 = Neutral, 4 = Agree, 5 = Strongly Agree)

Perceived Risk

9.1 I am concerned that using generative AI technology may pose data security or privacy risks.

9.2 I believe that using generative AI technology might negatively affect my teaching effectiveness.

9.3 I am worried that using generative AI technology may lead to technical failures or problems.

(1 = Strongly Disagree, 2 = Disagree, 3 = Neutral, 4 = Agree, 5 = Strongly Agree)

Behavioral Intention (BI)

10.1 I intend to use generative AI technology more in my future teaching.

10.2 I plan to regularly use generative AI technology in lesson preparation and implementation.

10.3 I am willing to learn more about generative AI technology and apply it to my teaching.

(1 = Strongly Disagree, 2 = Disagree, 3 = Neutral, 4 = Agree, 5 = Strongly Agree)

Use Behavior (UB)

11.1 I frequently use generative AI technology in my classroom teaching.

11.2 I already use generative AI technology in my daily lesson preparation or teaching.

11.3 I regularly use generative AI technology to enhance classroom interaction and teaching effectiveness.

(1 = Strongly Disagree, 2 = Disagree, 3 = Neutral, 4 = Agree, 5 = Strongly Agree)

**Supplementary Note 2: Informed Consent**

Dear Participants:

Hello! Thank you for your participation in this survey. This study aims to explore the willingness of pre-service music teachers to use AI. Your answers will provide us with valuable data and insights. Here, we hope that you can read the following information in detail and make a decision of whether to participate this survey based on full understanding.

**Voluntary participation:** Your participation is completely voluntary. You are free to choose whether to participate in this survey and can terminate the survey at any time without any adverse impact.

**Survey content:** The content of this survey is limited solely to the willingness of pre-service music teachers to use AI. All questions are related to the purpose of the research, and you do not have to worry about personal privacy leakage.

**Anonymity and confidentiality:** All answers of every participant will be strictly confidential, and the survey results will only be used for academic research and will be presented in the form of aggregated data without involving any personally identifiable information. Your personal information will not be disclosed or used for any commercial purpose.

**Data processing:** All collected data will be used for analysis of this study and will be used only for this study. We promise that your answers will not be used for other purposes.

**Informed consent:** By filling out this questionnaire, you indicate that you have fully understood the above content and voluntarily participated in this study.

If you have any questions about this study or need more information during your participation, please feel free to contact me (Name: Sirui He; Contact information: siruihe_@outlook.com).

Thank you again for your participation and your support!
